# The Genotype-Phenotype Association of Von Hipple Lindau Disease Based on Mutation Locations: A Retrospective Study of 577 Cases in a Chinese Population

**DOI:** 10.3389/fgene.2020.532588

**Published:** 2020-12-10

**Authors:** Jianhui Qiu, Kenan Zhang, Kaifang Ma, Jingcheng Zhou, Yanqing Gong, Lin Cai, Kan Gong

**Affiliations:** ^1^Department of Urology, Peking University First Hospital, Beijing, China; ^2^Institute of Urology, Peking University, Beijing, China; ^3^National Urological Cancer Center, Beijing, China

**Keywords:** Von Hippel-Lindau disease, genotype-phenotype, hypoxia-inducible factor α, tumor risk, elongin C

## Abstract

**Purpose:**

Von Hippel-Lindau (VHL) disease is a hereditary kidney cancer syndrome, with which patients are more likely to get affected by renal cell carcinoma (RCC), pancreatic cyst or tumor (PCT), central nervous system hemangioblastoma (CHB), retinal angiomas (RA), and pheochromocytoma (PHEO). Mutations of VHL gene located in 3p25 may impair the function of the VHL protein and lead to the disease. It’s unclear why obvious phenotype varieties exist among VHL patients. Here we aimed to ascertain whether the mutation types and locations affect the phenotype.

**Methods:**

We enrolled 577 Chinese VHL patients from 211 families and divided them into three groups and six subgroups according to their mutation types and locations. Cox survival analysis and Kaplan-Meier analysis were used to compare intergroup age-related tumor risks.

**Results:**

Patients with nonsense or frameshift mutations that were located before residues 117 of VHL protein (NoF1 subgroup) hold lower age-related risks of VHL associated tumors (*HR* = 0.638, 95%CI 0.461–0.883, *p* = 0.007), CHB (*HR* = 0.596, 95%CI 0.409–0.868, *p* = 0.007) or PCT (*HR* = 0.595, 95%CI 0.368–0.961, *p* = 0.034) than patients whose mutations were located after residues 117 (NoF2 subgroup). Patients in NoF1 subgroup still had lower age-related risks of CHB (*HR* = 0.652, 95%CI 0.476–0.893, *p* = 0.008) and PCT (*HR* = 0.605, 95%CI 0.398–0.918, *p* = 0.018) compared with those in combined NoF2 subgroup and other truncating mutation patients. NoF1 subgroup correspondingly had a longer estimated median lifespan (64 vs. 55 year, *p* = 0.037) than NoF2 subgroup. Among patients with missense mutations of VHL, only a small minority (23 of 286 missense mutations carriers) carried mutations involving neither HIF-α binding region nor elongin C binding region, who were grouped in MO subgroup. MO subgroup seemed to have a higher age-related risk of PHEO. In the whole cohort (*n* = 577), PHEO was an independent protective factor for CHB (*p* = 0.001) and survival (*p* = 0.005). RA and CHB failed to predict the age-related risk of each other.

**Conclusion:**

The mutation types and locations of VHL gene are associated with phenotypes. Genetic counselors could predict phenotypes more accurately based on more detailed genotype-phenotype correlations. Further genotype-phenotype studies should focus on the prediction of tumor recurrence, progression, and metastasis. The deep molecular mechanism of genotype-phenotype correlation is worth further exploring.

## Introduction

Von Hippel–Lindau (VHL) disease (OMIM 193300) is a hereditary kidney cancer syndrome that is an autosomal dominant disease caused by mutations of VHL gene, characterized by multiorgan and multicenter tumors, such as renal cell carcinoma (RCC), pancreatic cyst or tumor (PCT), central nervous system hemangioblastoma (CHB), retinal angiomas (RA), pheochromocytoma (PHEO), papillary cystadenoma of the broad ligament or epididymis, and endolymphatic sac tumor (Ong et al., 2007). VHL patients were diagnosed as type 1 when pheochromocytomas were absent and as type 2 when pheochromocytomas were proved (Haddad et al., 2013). CHB, RCC, RA, PCT, and PHEO are the most common symptoms of VHL disease (Nielsen et al., 2016; Carlo et al., 2019; Hong et al., 2019).

The VHL protein (pVHL), coded by the VHL gene located on 3p25, functions as an E3 ubiquitin protein ligase. The protein has 213 amino acids and consists of 2 domains: the α domain and the β domain. The best-characterized and well-known downstream pathway is that pVHL, elongin B and C, Cul2, and Rbx1 compose the pVHL complex that can bind and degrade transcription factor hypoxia-inducible factor α (actually HIF-1α, HIF-2α, HIF-3α; collectively HIF-α), which is an oxygen-dependent pathway. The hypoxia-inducible factor (HIF) transcription factor is composed by two subunits: the oxygen-dependent α subunit (1α, 2α, and 3α) and the constitutive expressing HIF1β subunit. Under the hypoxia condition or in the presence of the mutant VHL gene, the pVHL complex can’t recognize HIF-α so that HIF-α accumulates. Then it heterodimerizes with HIF1β and affects a group of downstream genes’ expression, including erythropoietin (EPO), vascular endothelial growth factor (VEGF), and transforming growth factor (TGF-α), to promote oncogenesis. Most of the pathogenic missense mutations are located in two regions of the VHL protein: the elongin C-binding site (residues 158–184 of the α domain) (Feldman et al., 1999; Ohh et al., 2000) and the HIF-α binding site (residues 65–117 of the β domain) (Hon et al., 2002; Lee et al., 2016).

There have been several studies focusing on genotype-phenotype correlations of VHL disease, providing some valuable tumor risk stratification methods mainly based on mutation types or locations of VHL gene. According to classical theories, type 2 VHL disease was due to missense mutations of VHL and truncating mutations were responsible for type 1 VHL disease (Ong et al., 2007). This theory was to be improved because even among patients with the same mutation types of the VHL gene, there existed phenotype diversities not explained clearly.

Several previous studies reported that significant phenotypic heterogeneity existed among missense mutations carriers (Hong et al., 2019). Mettu et al. (2010) collected phenotypic characters of 412 VHL patients with documented germline missense mutations and concluded that mutations in the α-domain of VHL protein were associated with a higher incidence of RA, compared with mutations in the β-domain. Liu et al. (2018) found that missense mutations located in HIF-binding region of VHL gene were associated with an increased risk of CHB and a decreased risk of PHEO. Forman et al. (2009) held the opinion that carriers of mutations involving the elongin C binding site were more likely to be affected by PEHO through structural analysis, compared with other missense mutations carriers. Only a few studies focused on internal phenotypic heterogeneity in truncating mutations. Ong et al. (2007) found nonsense or frameshift mutations were associated with earlier age of onset of RCC, higher age-related risks of RCC and RA than missense mutations or deletions. Large VHL gene deletions involving C3orf10 (HSPC300) were discovered to be related to a lower lifetime risk of RCC than deletions that do not involve C3orf10 (McNeill et al., 2009).

In this study, we regrouped VHL patients according to their mutation types: missense mutations group (M group), nonsense or frameshift mutations group (NoF group), and other truncating mutations that affect more than one nucleotide of VHL gene (oTR group). M group was further divided into HIF-α binding site mutations subgroup (MH subgroup), elongin C binding site mutations subgroup (ME subgroup), and mutations at other sites subgroup (MO subgroup). NoF group was seen as NoF2 subgroup if the mutations are located after residue 117 of VHL protein, otherwise as NoF1 subgroup. We aim to discover more detailed genotype-phenotype correlations through comparing the difference of age-related penetrance of VHL tumors and survival among subgroups.

## Materials and Methods

### Medical Ethics

This project had gained the approval of the Institutional Ethics Committee of Peking University First Hospital. Informed consents were signed by all patients or their legal guardians.

### Patient Recruitment and Assessment

A total of 577 Chinese VHL patients from 211 unrelated families diagnosed at Peking University First Hospital were recruited for this study. An individual was diagnosed with VHL disease when he or she carried a germline VHL mutation or met the clinical criteria for VHL disease (Shuin et al., 2006). Medical history and previous imaging data of all enrolled patients were collected. The age of onset of five main symptoms, which were CHB, RA, RCC, PCT, PHEO, were reviewed according to medical records, disease history, and imaging data of all VHL patients. Follow-up was carried out from the time genetic or clinically diagnosis of VHL disease for patients until death or January 1, 2019. Patients whose information was unavailable or inaccurate were removed from this cohort.

### Genetic Test of VHL Mutations

**FIGURE 1:**
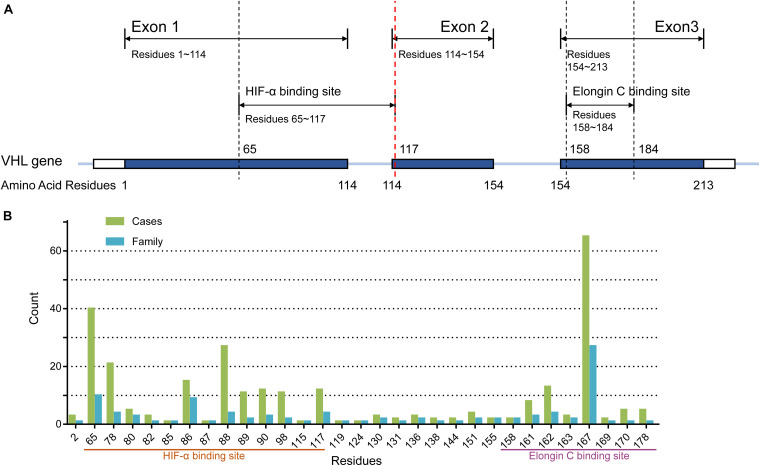
**(A)** The structure of the VHL gene. **(B)** The number of cases and families with missense mutations on each residues of pVHL.

Peripheral blood was drawn from each enrolled VHL patient unless he or she refused or was dead. DNA was extracted from all collected peripheral blood samples. The three exons of VHL and their flanking intronic sequences were amplified through polymerase chain reaction (PCR) and sequenced by Sanger sequencing. If a suspected patient was negative for PCR and Sanger sequencing, multiplex ligation-dependent probe amplification (MLPA, P016-C2 kit, MRC-Holland, Amsterdam, The Netherlands) followed by real-time quantitative PCR was used to detect large-scale mutations. Patients with point missense mutations were divided into MH subgroup (mutations in residues 65–117), ME subgroup (mutations in residues 158–184), and MO subgroup (mutations in other residues) (Figure 1A). Generally, truncating mutations were those mutations that can truncate or shorten the protein, such as nonsense mutations, frameshift mutations, splice-site mutations, and large-scale mutations. Patients with nonsense or frameshift mutations that were located at or before residues 117 of VHL protein were regrouped in NoF1 group, and others were regrouped in NoF2 subgroup. Other truncating mutations carriers were categorized in oTR group.

### Statistical Analysis

The Mann-Whitney *U*-test was used to compare the age at onset of each symptom. The survival time from birth to the end of follow-up or to death was used for survival analysis. The age-related risks of the five symptoms of VHL disease and survival analysis were calculated by Kaplan-Meier plot and log-rank analysis. And the Cox regression model was used for multivariate survival analysis to assess the effect of genotype on risk of the five symptoms. The correlation between two symptoms was analyzed by Chi-square (χ^2^) test. It’s considered to be statistically significant when *p*-value was less than 0.05. Data were analyzed by SPSS version 23.0 software (IBM, Chicago, IL).

## Results

### VHL Disease Patients’ Diagnosis and Enrollment

A total of 577 patients from 211 unrelated families diagnosed with the VHL disease in our center were enrolled in our retrospective study. One hundred families (286 patients), 60 families (165 patients), and 52 families (126 patients) were assigned to M group, NoF group, and oTR group, respectively (Table 1). For the 100 families in the M group, they carried a total of 50 mutations involving 42 gene loci or 31 residues of pVHL (Figure 1B). The median age at onset was 30 year (from 2 to 74 years), and the median lifespan of 131 dead patients was 42 years (from 11 to 71 year). Fifty-seven of 577 patients were diagnosed only by genetic tests but had not yet displayed any symptoms. Their age range was from 3 to 60 year, with a median of 15 year. Only five patients with pathogenic VHL mutations over the age of 40 year were asymptomatic. The five patients’ basic information is shown as Supplementary Table 1. Interestingly, some of the five patients’ direct relatives showed symptoms before their 40 year.

**TABLE 1:** The number of families and patients in each subgroup.

Group	Families/patients	Subgroup	Families/patients	Median age (year)	males/females	CHB (%)	RCC (%)	PEHO (%)	RA (%)	PCT (%)
M	100/286	MH	45/160	37.5	96/64	60.6	41.3	10.6	15.6	45.0
		ME	42/103	40	55/48	56.3	38.8	36.9	16.5	38.8
		MO	13/23	35	15/8	39.1	34.8	47.8	17.4	47.8
NoF	60/165	NoF1	32/85	39	55/30	64.7	38.8	9.4	24.7	32.9
		NoF2	28/80	40	41/39	76.3	55.0	7.5	21.3	52.5
oTR	52/126	–	–	38	67/59	72.2	48.4	5.6	23.8	50.0

*The morbidities of each disorder in every group were also calculated. M, Missense mutations; NoF, nonsense or frameshift mutations; oTR, truncating mutations except nonsense and frameshift mutations; MH, missense mutations in HIF-α binding site; ME, missense mutations in elongin C binding site; MO, missense mutations subgroup in other sites; NoF1, nonsense or frameshift mutations at or before residue 117 of VHL protein; NoF2, nonsense or frameshift mutations after residue 117 of VHL protein.*

### Differences in Age of Onset Among Subgroups

As mentioned before, among 520 patients who have shown clinical symptoms, the age at onset of VHL disease in our cohort ranged from 2 to 74 year, with a median of 30 year. CHB was the first symptom of more than half of the patients (311 of 520, 59.8%). Even in each subgroup, CHB was still the most common first symptom. The median age of onset of CHB, RA, RCC, PCT, PHEO is 31 year (3–66 year), 26 year (2–66 year), 37 year (14–74 year), 33 year (14–67 year), 37 year (9–68 year). The number of patients affected by each symptom was shown as Supplementary Table 2. There was no significant distinction in the age of onset between males and females (30 vs. 30.5 year, *p* = 0.748). NoF1 subgroup had a later age of onset than NoF2 (median age of onset, 28 vs. 32 year, *p* = 0.018). Significant differences in age of onset were not discovered among subgroup MH, ME, and MO (*p* = 0.42). There was no significant difference between group NoF and oTR or between group M and those in the combined NoF and oTR together, either (*p* = 0.983 and *p* = 0.065, respectively).

Pathologically, RA and CHB were all hemangioblastomas. But in the whole cohort (*n* = 577) the onset of the two symptoms did not appear to be related (χ^2^ = 0.138, *p* = 0.711). Patients with CHB didn’t show higher age-related risks of RA (*p* = 0.494, Supplementary Figure 1A), and patients with RA weren’t associated with higher age-related risks of CHB (*p* = 0.055, Supplementary Figure 1B).

### Comparison of Age-Related Tumor Risk Among Subgroup

**FIGURE 2:**
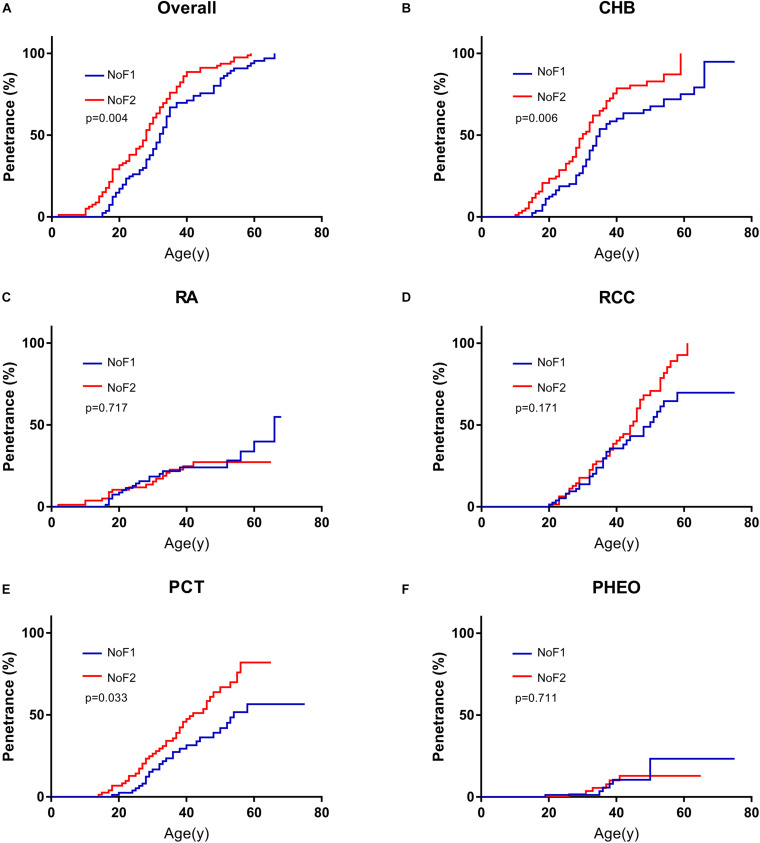
Comparison of age-related risks in NoF1 and NoF2 subgroup. **(A)** All the five VHL-associated tumors, **(B)** CHB, **(C)** RA, **(D)** RCC, **(E)** PCT, **(F)** PHEO. NoF1, nonsense or frameshift mutations before residue 117 of VHL protein; NoF2, nonsense or frameshift mutations after residue 117 of VHL protein; CHB, central nervous system hemangioblastoma; RA, retinal angiomas; RCC, renal cell carcinoma; PCT, pancreatic cyst or tumor; PHEO, pheochromocytoma.

Because of the significant difference in age of onset between subgroup NoF1 and NoF2, we compared these two subgroups further. NoF1 subgroup took a lower age-related risk of VHL-associated tumors than subgroup NoF2 (*p* = 0.004). NoF1 subgroup had lower age-related risks of PCT (*p* = 0.033) and CHB (*p* = 0.006) compared with NoF2, but risks of RA (*p* = 0.717), RCC (*p* = 0.171), and PHEO (*p* = 0.711) were similar between the two subgroups (Figure 2). Accordingly, multivariate Cox regression analysis revealed that NoF1 mutations were independently associated with a lower risk of VHL-associated tumors (*HR* = 0.689, 95%CI 0.497–0.953, *p* = 0.025), CHB (*HR* = 0.596, 95%CI 0.409–0.868, *p* = 0.007), and PCT (*HR* = 0.595, 95%CI 0.368–0.961, *p* = 0.034) compared with NoF2 mutations (Table 2).

**TABLE 2:** Univariate and multivariate Cox regression analysis of age-related VHL tumor risks of NoF1 vs. NoF2.

Tumor	Variables	Univariate analysis			Multivariate analysis		
		HR	95% CI	*p*-value	HR	95% CI	*p*-value
Overall	Sex (Male vs. Female)	0.827	0.599–1.143	0.251	0.853	0.617–1.179	0.334
	Mutation (NoF1 vs. NoF2)	0.632	0.457–0.874	**0.005**	0.638	0.461–0.883	**0.007**
CHB	Sex (Male vs. Female)	1.122	0.773–1.629	0.546	1.155	0.795–1.678	0.450
	Mutation (NoF1 vs. NoF2)	0.601	0.412–0.875	**0.008**	0.596	0.409–0.868	**0.007**
RCC	Sex (Male vs. Female)	0.962	0.606–1.527	0.869	0.944	0.594–1.501	0.808
	Mutation (NoF1 vs. NoF2)	0.733	0.467–1.153	0.179	0.731	0.465–1.150	0.176
PCT	Sex (Male vs. Female)	0.801	0.499–1.284	0.356	0.788	0.491–1.264	0.323
	Mutation (NoF1 vs. NoF2)	0.600	0.371–0.968	**0.036**	0.595	0.368–0.961	**0.034**
PHEO	Sex (Male vs. Female)	0.650	0.228–1.855	0.421	0.657	0.230–1.878	0.433
	Mutation (NoF1 vs. NoF2)	1.222	0.423–3.534	0.711	1.193	0.411–3.459	0.745
RA	Sex (Male vs. Female)	0.827	0.435–1.570	0.561	0.823	0.433–1.564	0.552
	Mutation (NoF1 vs. NoF2)	1.125	0.592–2.139	0.718	1.133	0.596–2.155	0.703

*CI, confidence interval; HR, hazard ratio; NoF1, nonsense or frameshift mutations before residue 117 of VHL protein; NoF2, nonsense or frameshift mutations after residue 117 of VHL protein; CHB, central nervous system hemangioblastoma; RA, retinal angiomas; RCC, renal cell carcinoma; PCT, pancreatic cyst or tumor; PHEO, pheochromocytoma. If p < 0.05, the p-value will be bold.*

Nonsense mutations, frameshift mutations were often regarded as truncating mutations in previous studies (Ong et al., 2007; Forman et al., 2009; Liu et al., 2018). Through comparisons in the age of onset among subgroups, it was proven that there were some differences in truncating mutations. Age-related risks of overall VHL-associated tumors or each VHL tumor were similar between NoF2 subgroup and oTR group. So, we combined patients in NoF2 subgroup and oTR group into one group (N2TR) and compared the group with NoF1 subgroup. NoF1 subgroup was related to lower risks of overall VHL-associated tumors (*p* = 0.038), CHB (*p* = 0.006), PCT (*p* = 0.022) compared with N2TR group. Meanwhile, the risks of RA, RCC, or PHEO didn’t bring out significant differences between the two groups (Supplementary Figure 2). Correspondingly, multivariate Cox regression analysis revealed that compared with the N2TR group, NoF1 subgroup was the protective factor of CHB (*HR* = 0.652, 95% CI 0.476–0.893, *p* = 0.008) and PCT (*HR* = 0.605, 95% CI 0.398–0.918, *p* = 0.018) (Table 3). It’s worth noting that males were associated with a lower risk of PCT (*HR* = 0.654, 95% CI 0.465–0.919, *p* = 0.015) in patients with truncating mutations.

**TABLE 3:** Univariate and multivariate Cox regression analysis of age-related VHL tumor risks of NoF1 vs. N2TR.

Tumor	Variables	Univariate analysis			Multivariate analysis		
		HR	95% CI	*p*-value	HR	95% CI	*p*-value
Overall	Sex (Male vs. Female)	0.852	0.667–1.090	0.203	0.869	0.679–1.113	0.266
	Mutation (NoF1 vs. N2TR)	0.758	0.578–0.993	**0.045**	0.767	0.584–1.006	0.055
CHB	Sex (Male vs. Female)	0.981	0.745–1.292	0.893	0.998	0.758–1.315	0.991
	Mutation (NoF1 vs. N2TR)	0.652	0.476–0.893	**0.008**	0.652	0.476–0.893	**0.008**
RCC	Sex (Male vs. Female)	0.964	0.687–1.353	0.833	0.966	0.688–1.356	0.841
	Mutation (NoF1 vs. N2TR)	0.768	0.519–1.137	0.187	0.768	0.519–1.137	0.188
PCT	Sex (Male vs. Female)	0.653	0.464–0.917	**0.014**	0.654	0.465–0.919	**0.015**
	Mutation (NoF1 vs. N2TR)	0.604	0.398–0.916	**0.018**	0.605	0.398–0.918	**0.018**
PHEO	Sex (Male vs. Female)	0.741	0.315–1.746	0.493	0.747	0.317–1.761	0.506
	Mutation (NoF1 vs. N2TR)	1.395	0.577–3.371	0.460	1.384	0.572–3.346	0.471
RA	Sex (Male vs. Female)	1.180	0.725–1.919	0.505	1.177	0.723–1.917	0.513
	Mutation (NoF1 vs. N2TR)	1.054	0.629–1.766	0.841	1.042	0.621–1.747	0.876

*CI, confidence interval; HR, hazard ratio; NoF1, nonsense or frameshift mutations before residue 117 of VHL protein; N2TR, mutations in combined with nonsense or frameshift mutations after residue 117 of VHL protein and other truncating mutations; CHB, central nervous system hemangioblastoma; RA, retinal angiomas; RCC, renal cell carcinoma; PCT, pancreatic cyst or tumor; PHEO, pheochromocytoma. If p < 0.05, the p-value will be bold.*

**FIGURE 3:**
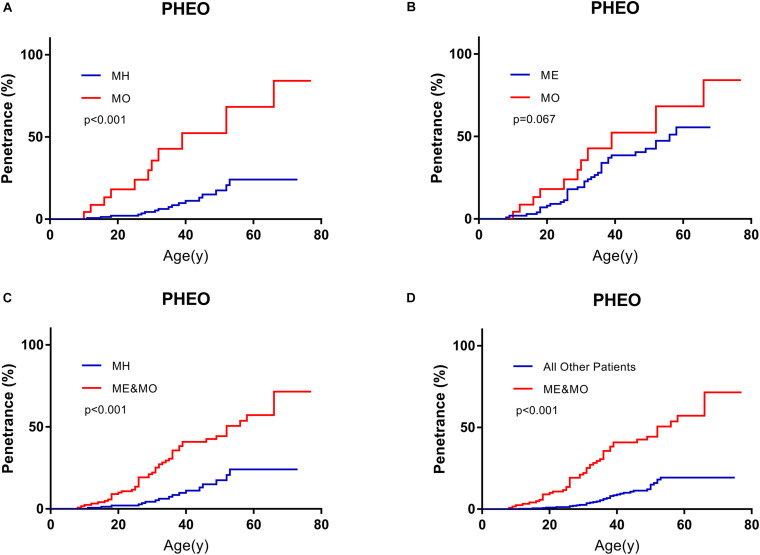
Comparison of age-related risks of PHEO between MO subgroup and MH subgroup **(A)**, MO subgroup and ME subgroup **(B)**, MH subgroup and those in combination with MO and ME subgroups **(C)**, those in combined with MO and ME subgroups and all other VHL patients **(D)**. PHEO, pheochromocytoma; MH, missense mutations in HIF-α binding site; ME, missense mutations in elongin C binding site; MO, missense mutations subgroup in other sites.

On the basis of predecessors, we classified patients with missense mutations (M group) by mutation locations into three subgroups, whose missense mutations were located in the HIF-binding region (residues 65–117, MH subgroup), Elongin C binding region (residues 158–184, ME subgroup), and others region (MO subgroup), respectively (Ong et al., 2007; Lee et al., 2016; Liu et al., 2018; Salama et al., 2019). It’s a remarkable fact that the number of MO group was far less than the other two groups in our cohort. Compared with MH subgroup and ME subgroup, patients in MO subgroup seemed to be more likely to have PHEO (*p* < 0.001, compared with MH subgroup, Figure 3A; *p* = 0.067, compared with ME subgroup, Figure 3B). Though age-associated risks of PHEO between MO and ME subgroup didn’t show significant differences (*p* = 0.067), it may be due to the limited sample size of MO subgroup. If we regarded ME and MO as a whole subgroup, this subgroup had a higher penetrance of PHEO than MH subgroup (*p* < 0.001, Figure 3C), or than those in combined all other patients (*p* < 0.001, Figure 3D). No significant difference in age-related risks of other symptoms was discovered among the three M subgroups.

We diagnosed patients affected by PHEO before death or the end of follow-up as type 2 VHL disease and those not affected by PHEO as type 1 VHL disease. Finally, 87 of 577 patients were diagnosed as type 2 VHL disease. Type 1 patients were associated with higher risks of CHB (*p* = 0.001), RA (*p* = 0.051), and lower risks of RCC (*p* = 0.053) than those with type 2 disease. The risks of VHL-associated tumors (*p* = 0.068) or PCT (*p* = 0.083) between the two types didn’t show a significant difference. We could then come to the conclusion that appearance of PHEO was a protective factor for CHB, and its effect on RA, RCC was still unclear (Supplementary Figure 3).

It’s generally acknowledged that residues prior to 113 of VHL protein were coded by exon 1, residues 115–153 by exon 2, and residues after 155 by exon 3 (Gnarra et al., 1996; Hansen et al., 2002). Patients in M group with mutations in exon 2 vs. exons 1 or 3 had a higher age-related risk of PCT on univariate (*HR* = 2.045, 95%CI 1.249–3.348, *p* = 0.004) and multivariate analyses (*HR* = 2.053, 95%CI 1.253–3.362, *p* = 0.004; Table 4).

**TABLE 4:** The univariate and multivariate cox analyses of age-related risks of PCT.

Variables	Univariate analysis			Multivariate analysis		
	HR	95% CI	*p*-value	HR	95% CI	*p*-value
Sex (Male vs. Female)	0.769	0.602–0.983	**0.036**	0.925	0.647–1.324	0.671
Mutations (exon 2 vs. exon 1 or 3)	2.045	1.249–3.348	**0.004**	0.487	0.297–0.798	**0.004**

*PCT, pancreatic cyst or tumor; CI, confidence interval; HR, hazard ratio. If p < 0.05, the p-value will be bold.*

### Difference in Survival Between Groups

**FIGURE 4:**
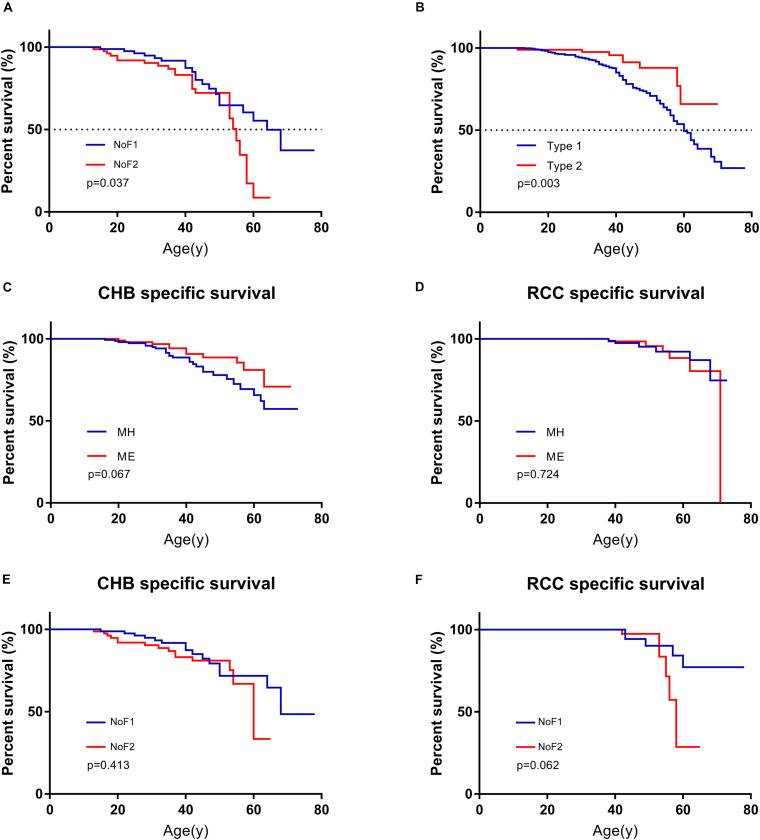
Comparison of survival in patients with different mutation groups. **(A)** Comparison of survival in patients from NoF1 and NoF2 subgroups. **(B)** Comparison of survival in type 1 and type 2 patients. **(C)** Comparison of CHB-specific survival in patients with MH and ME mutations. **(D)** Comparison of RCC-specific survival in patients with MH and ME mutations. **(E)** Comparison of CHB-specific survival in patients with NoF1 and NoF2 mutations. **(F)** Comparison of RCC-specific survival in patients with NoF1 and NoF2 mutations. NoF1, nonsense or frameshift mutations before residue 117 of VHL protein; NoF2, nonsense or frameshift mutations after residue 117 of VHL protein; CHB, central nervous system hemangioblastoma; MH, missense mutations in HIF-α binding site; ME, missense mutations in elongin C binding site; RCC, renal cell carcinoma.

The estimated median lifespan of all 577 patients was 62 year. Because MO subgroup was too small and all of them were alive, we didn’t have access to their survival data. The differences between the median survival of MH subgroup and ME subgroup weren’t statistically significant (62 vs. 63 year, *p* = 0.259). Missense mutations carriers seemed to have longer estimated median survival than others (63 vs. 58 year), but the difference was not statistically significant (*p* = 0.090). The estimated median survival of patients in NoF1 subgroup was 9 years longer than those in NoF2 subgroup (64 vs. 55 year, *p* = 0.037, Figure 4). A NoF2 mutation was an independent risk factor for VHL patients’ survival in comparison with NoF1 mutations on univariate (*HR* = 1.900, 1.024–3.525, *p* = 0.042) and multivariate (*HR* = 1.888, 95%CI 1.017–3.506, *p* = 0.044) Cox regression analysis (Supplementary Table 3). Type 2 patients had significantly better survival than type 1 patients (*p* = 0.005, Figure 4B). Type 2 disease was an independent protective factor of survival, compared with type 1 patients (*HR* = 0.361, 95%CI 0.177–0.740, *p* = 0.005, Supplementary Table 3).

### CHB and RCC Specific Survival Among Subgroups

A total of 131 of 577 patients had died of some causes, which were categorized as CHB related, RCC related, and other causes of death. CHB and RCC were the most common death causes of VHL patients, which were shown as Supplementary Table 4. A total of 90.8% (119 of 131) of deaths were VHL related. Their death age ranged from 11 to 71 with a median of 42. As mentioned before, we didn’t have access to survival data of patients in the MO subgroup. We failed to find differences between MH subgroup and ME subgroup for CHB- or RCC-specific survival (*p* = 0.067 and *p* = 0.724, respectively). There was no difference for CHB or RCC specific survival between NoF1 and NoF2 subgroups (*p* = 0.413 and *p* = 0.062, respectively, Figure 4).

## Discussion

The VHL disease is characterized by lifelong multiorgan and multisite tumors risks. Genotype-phenotype correlations are crucial to clinical genetic counseling. Several previous studies have focused on genotype-phenotype correlations of the VHL disease, but there was still a lack of authoritative consensus (Ong et al., 2007; Mettu et al., 2010; Lee et al., 2016; Lomte et al., 2018). The typical classification methods divide VHL patients into type 1 and type 2 based on the penetrance of PHEO. In our study, type 1 patients were associated with higher lifetime risks of CHB and worse survival compared with type 2 patients. These results suggested that type 1 or 2 VHL disease classification was an easy but rough way of risk stratification in clinicians’ daily work.

Our study suggests that MO subgroup should be treated as a separate subgroup independent of ME and MH subgroup, and NoF1 subgroup should be seen as a separate subgroup with a relatively better prognosis independent of the N2TR subgroup. These discoveries have the potential to improve and detail clinical counseling of VHL disease and reveal the underlying molecular mechanisms.

In our cohort, we subdivided the M group into MH, ME, and MO subgroups. The age-related risks of PHEO in MO and ME subgroups were higher than in other patients. MO subgroup seemed to be more likely to be affected by PHEO than MH subgroup, but no significant difference was discovered. The small number of people in MO subgroup and not long enough follow-up time limited more detailed genotype-phenotype research. There wasn’t significant difference in lifespan, CHB, or RCC specific survival between MH and ME subgroup. This conclusion wasn’t consistent with some previous papers, which held the opinions that MH subgroup had worse CHB specific survival compared with other missense mutations carriers (Lee et al., 2016; Liu et al., 2018). It might explain this divergence that we defined the new MO subgroup from non-MH missense mutations carriers and we expanded the sample size. However, there is a lack of evidence at molecular levels to support the rationality of this classification method, which is mainly based on VHL-HIF pathway. VHL protein is able to interact with several other molecular pathways involving a diverse array of genes’ transcription, deposition of the extracellular matrix, and the regulation of the microtubule cytoskeleton (Czyzyk-Krzeska and Meller, 2004; Frew and Krek, 2008; Xue et al., 2012). *In vitro* and *in vivo* studies revealed that some mutations involved in type 2 VHL disease retained the ability to regulate HIF-α subunits, suggesting that there must be more underlying mechanisms that are responsible for VHL disease (Clifford et al., 2001; Hoffman et al., 2001). In other words, there are several pathways besides the VHL-HIF pathway through which the mutant VHL gene leads to the disease (Roe et al., 2006; Forman et al., 2009). So, the classification of mutations based on the VHL-HIF pathway is not suitable for all VHL-associated phenotypes. Mutations at different locations are likely to change the intrinsic structure and interfaces of the VHL protein (Minervini et al., 2019), resulting in deregulation of the corresponding pathways and tumorigenesis. Further studies should classify mutations based on their effects on the structure and functions of the VHL protein rather than merely on the basis of mutation locations.

Ong et al. (2007) had made nonsense and frameshift mutations and deletions as two independent types of mutations and found nonsense and frameshift mutations were associated with higher lifetime risks of RCC. In our cohort, the NoF group was divided into NoF1 and NoF2 subgroups to explore phenotype heterogeneity in NoF group. NoF1 subgroup had lower age-related risks of overall VHL-associated tumors, CHB, and PCT than N2TR subgroup and longer estimated lifespans than NoF2 subgroup.

Although RA and CHB all belonged to hemangioblastomas, one’s appearance failed to remind higher lifetime risks of the other.

According to our conclusion, it’s consistent with previous studies that CHB is the most common symptom and CHB and RCC are the lead causes of death (Binderup et al., 2017). On this account, the management of VHL disease should focus on the control of CHB and RCC. In our cohort, the estimated median lifespan of all patients is 62 year. There isn’t access to survival information of MO subgroup.

For PCT, patients in M group with mutations in exon 2 vs. exons 1 or 3 were under higher age-related risks. This conclusion is inconsistent with Tirosh et al. (2018) whose work supported mutations in exon 3 were more likely affected by pancreatic neuroendocrine tumors (PNETs). Perhaps it’s because the definition of PCT is different from the definition of PNETs. In oTR group, males had a lower risk of PCT than females. In view of the divergences and uncertainties, more research is needed to explore the risk factors of PCT in VHL patients.

At present, the main management measures of VHL disease are active surveillance and surgical intervention when necessary. Clinicians need to weigh the risks of tumor progression and metastasis against the risks of surgical intervention and complication. There is a lack of scientific and authoritative surgical indications for each of five main symptoms to refer to. Existing standards often focus mainly on clinical parameters but ignore mutation types of patients. VHL associated endolymphatic sac tumors (ELSTs) are potential to cause audio vestibular morbidity even when an ELST mass can’t be identified on imaging examinations. So, excision is suggested as soon as an ELST or ELST associated morbidity is discovered. VHL associated CHB are usually multisite, of which natural history is still unpredictable. According to the current mainstream view, only symptomatic tumors need to be resected (Dornbos et al., 2018). It’s generally recognized that RCC of VHL patients whose longest diameter is below 3 cm don’t need surgical interventions (Duffey et al., 2004; Carlo et al., 2019). Peng et al. (2019) held the opinion that the criterion should be relaxed up to 4 cm. PNETs of VHL patients were considered not at risk of metastasis with a diameter below 1.2 cm and high risks of malignancy and metastasis with a diameter greater than 3 cm. For patients with a PNET diameter between 1.2 and 3 cm, only those with VHL missense mutations were observed to develop metastasis (Tirosh et al., 2018). However, these results aren’t enough to make an accepted criterion for PNET surgical intervention. In the following studies, the VHL genotype should be taken into consideration to determine whether surgical treatments are needed or not, to predict the risk of tumor recurrence, progression, and metastasis, and to predict therapeutic effects of non-surgical interventions.

In this study, we describe a modified genotype-phenotype correlation of VHL disease on the basis of previous studies, according to the VHL-HIF pathway. The correlation tries to subgroup truncating mutations and missense mutations, predicting tumor penetrance and survival of VHL patients. Our results will work as a VHL disease risk stratification method and provide more detailed information for genetic counseling.

## Data Availability Statement

The datasets for this article are not publicly available because we have a responsibility to protect patients’ privacy. Requests to access the datasets should be directed to KG, gongkan_pku@126.com.

## Ethics Statement

The studies involving human participants were reviewed and approved by the Institutional Ethics Committee of the Peking University First Hospital. Written informed consent to participate in this study was provided by all patients or their legal guardians.

## Author Contributions

JQ: conceptualization. KM and JZ: data curation. JQ and KZ: formal analysis and original draft writing. KG: funding acquisition. KG, YG, and LC: methodology and draft review and revision. KG and LC: project administration and supervision. All authors contributed to the article and approved the submitted version.

## Conflict of Interest

The authors declare that the research was conducted in the absence of any commercial or financial relationships that could be construed as a potential conflict of interest.
