# Supplementary material for: The Genotype-Phenotype Association of Von Hipple Lindau Disease Based on Mutation Locations: A Retrospective Study of 577 Cases in a Chinese Population
Source: Front Genet. 2020 Dec 10;11:532588. doi: 10.3389/fgene.2020.532588 (PMC7762453; doi:10.3389/fgene.2020.532588)
Supplement: Supplementary file 1 [file Data_Sheet_1.docx]

Supplementary Material

# Supplementary Data

None.

# Supplementary Figures and Tables

## Supplementary Figures


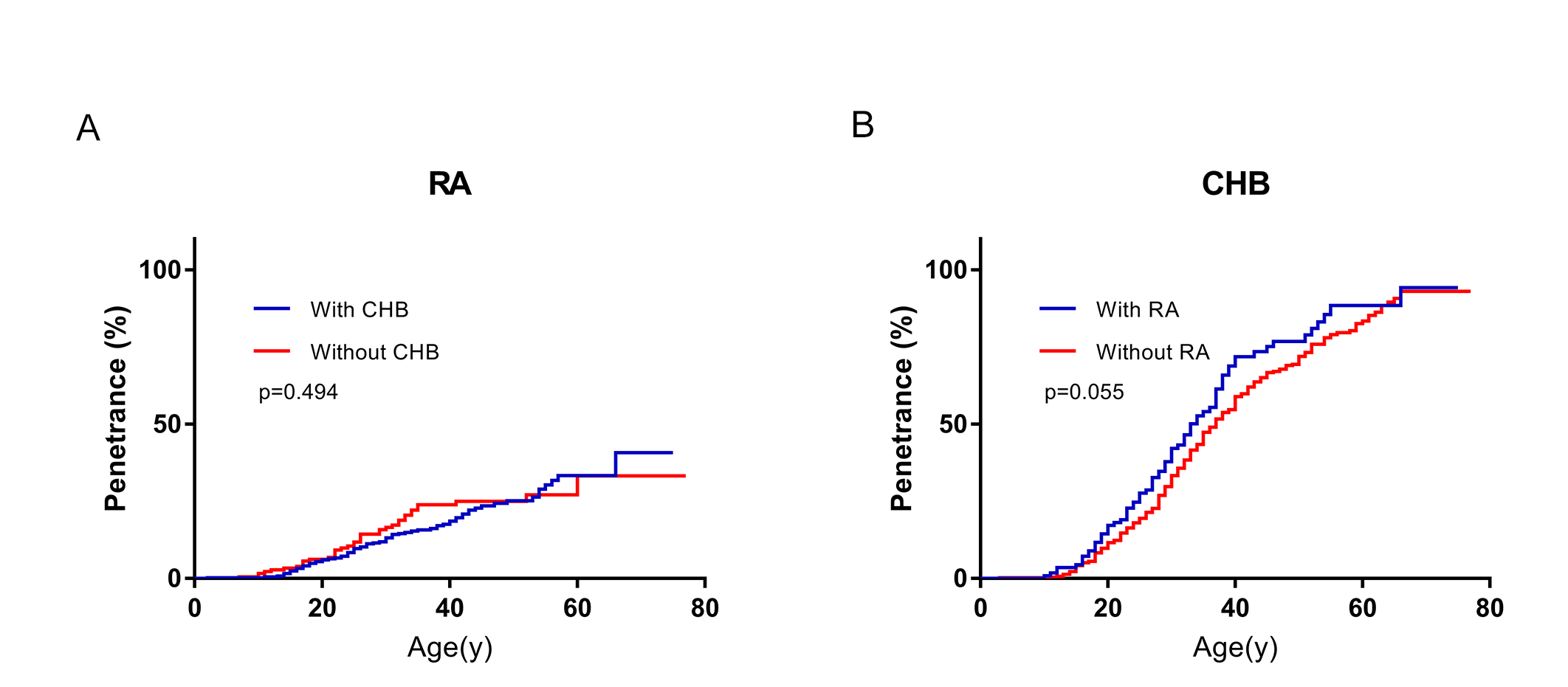


**Supplementary Figure 1.** The correlation between CHB and RA. Comparison of age-related risk of RA between patients with and without CHB **(A)**. Comparison of age-related risk of CHB between patients with and without RA **(B)**. CHB, central nervous system hemangioblastoma; RA, retinal angioma.


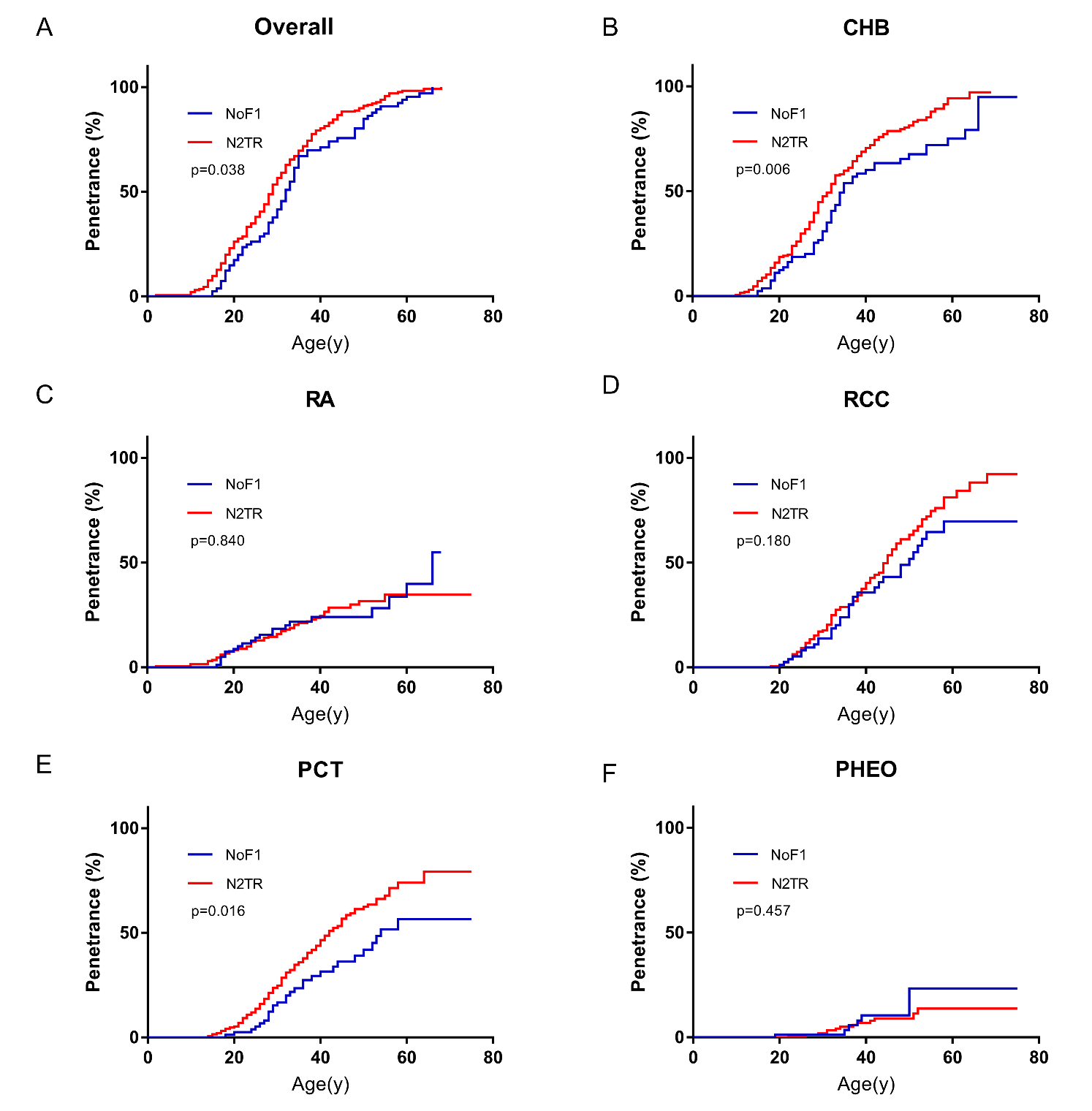


**Supplementary Figure 2.** Comparison of age-related risks in NoF1 and N2TR subgroup. **(A)** VHL-associated tumors, **(B)** CHB, **(C)** RA, **(D)** RCC, **(E)** PCT, **(F)** PHEO. NoF1, nonsense or frameshift mutations before residue 117 of VHL protein; N2TR, mutations in combined with nonsense or frameshift mutations after residue 117 of VHL protein and other truncating mutations; CHB, central nervous system hemangioblastoma; RA, retinal angiomas; RCC, renal cell carcinoma; PCT, pancreatic cyst or tumor; PHEO, pheochromocytoma.


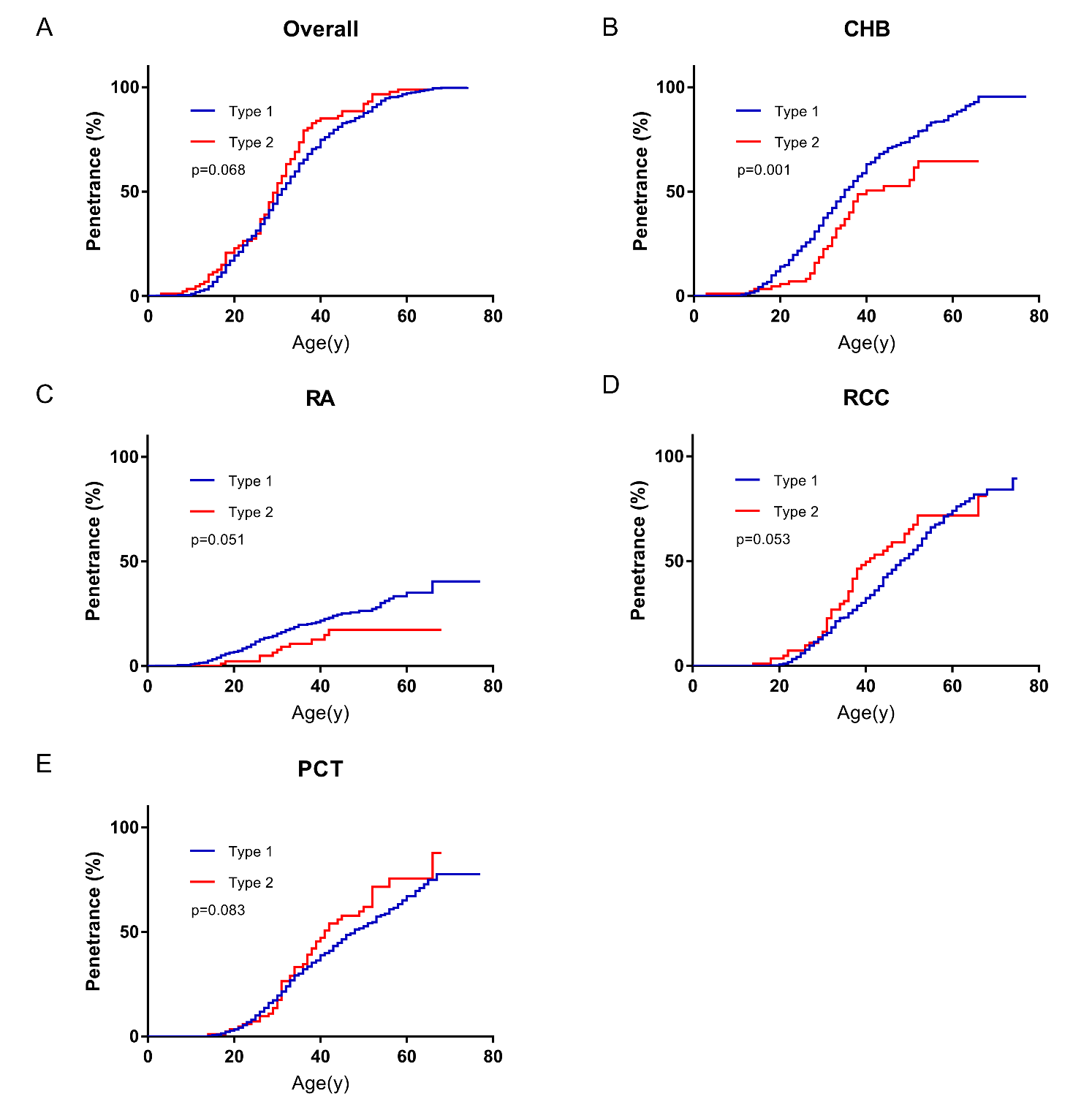


**Supplementary Figure 3.** Comparison of age-related risks in type 1 and 2 patients. **(A)** VHL-associated tumors, **(B)** CHB, **(C)** RA, **(D)** RCC, **(E)** PCT. CHB, central nervous system hemangioblastoma; RA, retinal angiomas; RCC, renal cell carcinoma; PCT, pancreatic cyst or tumor.

## Supplementary Tables

**Supplementary Table 1.** The 5 patients who had not yet show symptoms until the end of follow-up.

| Patient ID | mutation | subgroup | Ask age |
| --- | --- | --- | --- |
| 77 | Exon 1 deletion | oTR | 50 |
| 267 | Exon 1 deletion | oTR | 60 |
| 283 | c.499C>T | ME | 55 |
| 574 | c.224delT | NoF1 | 43 |
| 629 | Exon 1, 2, 3 deletion | oTR | 51 |

oTR, truncating mutations except nonsense and frameshift mutations; ME, missense mutations in elongin C binding site; NoF1, nonsense or frameshift mutations at or before residue 117 of VHL protein.

**Supplementary Table 2.** Median age of onset of 5 main symptoms and the number of patients affected by the 5 symptoms.

|  | Median age of onset | Male | Female | Total |
| --- | --- | --- | --- | --- |
| CHB | 31 (3 to 66) | 220 | 151 | 371 |
| RA | 26 (2 to 66) | 69 | 45 | 114 |
| RCC | 37 (14 to 74) | 153 | 99 | 252 |
| PCT | 33 (14 to 67) | 136 | 120 | 256 |
| PHEO | 37 (9 to 68) | 54 | 33 | 87 |

CHB, central nervous system hemangioblastoma; RA, retinal angiomas; RCC, renal cell carcinoma; PCT, pancreatic cyst or tumor; PHEO, pheochromocytoma

**Supplementary Table 3.** The univariate and multivariate Cox analyses of overall survival in VHL patients

| Variables | Univariate analysis | | | Multivariate analysis | | |
| --- | --- | --- | --- | --- | --- | --- |
|  | HR | 95% CI | p value | HR | 95% CI | p value |
| Sex (Male vs Female) | 1.134 | 0.614-2.094 | 0.688 | 1.094 | 0.591-2.029 | 0.774 |
| Mutation (NoF2 vs NoF1) | 1.900 | 1.024-3.525 | **0.042** | 1.888 | 1.017-3.506 | **0.044** |
| Sex (Male vs Female) | 0.989 | 0.699-1.400 | 0.952 | 0.992 | 0.701-1.403 | 0.962 |
| Type (Type 2 vs 1) | 0.361 | 0.177-0.740 | **0.005** | 0.361 | 0.177-0.740 | **0.005** |

CI, confidence interval; HR, hazard ratio; NoF1, nonsense or frameshift mutations before residue 117 of VHL protein; NoF2, nonsense or frameshift mutations after residue 117 of VHL protein.

**Supplementary Table 4.** The death causes of 131 departed patients

|  | CHB | | | RCC | | | Other causes | | |
| --- | --- | --- | --- | --- | --- | --- | --- | --- | --- |
| Sex | NO | Median death age | Death age range | NO | Median death age | Death age range | NO | Median death age | Death age range |
| Male | 45 | 37 | 15-68 | 22 | 57 | 38-71 | 9 | 43 | 11-62 |
| Female | 41 | 37 | 13-63 | 11 | 49 | 38-56 | 3 | 45 | 42-47 |
| Total | 86 | 37 | 13-68 | 33 | 53 | 38-71 | 12 | 44 | 11-62 |

CHB, central nervous system hemangioblastoma; RCC, renal cell carcinoma.
